# Are Differences in the Oral Microbiome Due to Ancestry or Socioeconomics?

**DOI:** 10.1128/mSystems.00836-19

**Published:** 2020-03-10

**Authors:** Christopher Kenyon

**Affiliations:** a Department of Clinical Science, Institute of Tropical Medicine, Antwerp, Belgium; Qingdao Institute of Bioenergy and Bioprocess Technology, Chinese Academy of Sciences

**Keywords:** oral microbiome, ancestry

## LETTER

In their recent paper, Yang et al. found differences between European Americans (EAs) and African Americans (AAs) in the abundance of various oral bacterial species based on 16S rRNA sequencing of a cohort from the southeastern United States (1). They concluded that these differences are due to intrinsic ancestry-based differences in oral microbiomes. I would advocate caution in reaching this conclusion based on the small differences identified and the concern that these differences may be due to inadequate controlling for socioeconomic factors. The differences in relative abundance of the 13 most common taxa between EA and AA were small (batch 1, median 1.2% [interquartile range {IQR}, 0.5 to 4%]; batch 2, median 0.2% [IQR, 0.2 to 0.9%]). There were large and statistically significant differences in age, gender, income, highest level of education, smoking, and proportion of participants with tooth loss between the two groups. While some of these differences were controlled for in the analyses, it is quite possible that they were inadequately controlled for. For example, only 13.1% of AAs versus 25.1% of EAs retained all of their teeth. A greater proportion of AAs had lost 1 to 10 and more than 10 teeth. This is a crude measure of periodontal health. Controlling with a more detailed measure of periodontal health may have explained most or all the differences found. Dental caries have been found to be more common in AAs than EAs and to be strongly associated with lower income and poorer education (2–4). The finding by Yang et al. that bacterial taxa that are well-known causes of periodontal disease, such as Porphyromonas gingivalis, Prevotella intermedia, Treponema denticola, and Filifactor alocis, were more prevalent in AAs than EAs is commensurate with this socioeconomic explanation.
